# Efficacy of the photobiomodulation therapy in the treatment of the burning mouth syndrome

**DOI:** 10.4317/medoral.23143

**Published:** 2019-10-27

**Authors:** Elena Bardellini, Francesca Amadori, Giulio Conti, Alessandra Majorana

**Affiliations:** 1 University of Brescia, Department of Oral Medicine, Dental Clinic, P.le Spedali Civili di Brescia, 25133 Brescia, Italy; 2 University Vita-Salute S. Raffaele, 10090 Milan, Italy

## Abstract

**Background:**

This study aims to evaluate the efficacy of the photobiomodulation therapy (PBMT) - in terms of pain and of quality of life- in patients affected by burning mouth syndrome (BMS).

**Material and Methods:**

This study was designed as a randomised double-blinded prospective study. Patients diagnosed with BMS in the period from June 2015 to June 2018 were recruited. The patients were randomised into two groups and each received treatment once a week for ten weeks: group A received laser therapy (K Laser Cube 3®) while group B was given sham therapy (placebo). Pain was evaluated through the Visual Analogue Scale (VAS) and quality of life was assessed with the short form of the Oral Health Impact Profile (OHIP-14). Assessment was done at baseline and after every therapy session. The researchers were blind to the randomised allocations.

**Results:**

A total of 85 patients were analysed. Group A (laser treatment) was composed of 43 patients while group B (sham therapy) of 42 patients. Patients treated with PBMT showed a significant decrease in symptoms (*p*=0.0008) and improved quality of life related to oral health (*p*=0.0002).

**Conclusions:**

PBMT has demonstrated to have a positive effect in relieving BMS symptoms and in improving a patient’s overall quality of life.

** Key words:**Burning mouth syndrome, oral cavity, lasers, life quality.

## Introduction

Burning Mouth Syndrome (BMS) is a complex disorder characterised by a burning or stinging sensation in the oral mucosa without any detecTablechanges upon physical examination. This disorder is sometimes accompanied by dysgeusia and xerostomia (1). The continuous sensation of burning or heat usually affects the tongue (particularly the tip and lateral borders), but it can also affect the lips or the hard/soft palate (2-3). BMS most commonly occurs in patients over the age of 50, with a male-female ratio of 1:7; its reported prevalence from several international studies varies from 0.7 to 4.6% (4). The aetiology of BMS can be attributed to numerous local and systemic factors (5); such local factors include parafunctional habits, badly fitting prostheses, allergic reactions, infections, taste alterations and xerostomia. Associated systemic factors are: endocrine alterations (hypothyroidism, diabetes, and the menopause), vitamin B complex, iron and zinc deficiencies, anaemia, gastro esophageal reflux and Sjogren’s syndrome have been studied (5). For several years, BMS was also attributed to psychological factors, such as anxiety, depression and psychological stress (6). In addition, recent studies (7-9) have explored the possible pathophysiological contribution of neuropathic mechanisms acting on different levels of the neuraxis (2).

Being a multifactorial disease, the treatment or elimination of a systemic, local or psychological factor often determines improvement of BMS pain and symptoms. If causal therapy does not work, symptom management varies from topical treatment- i.e. epithelial protector, capsaicin, aloe vera, topical clonazepam and lidocaine (10-12) - to systemic drugs, including systemic capsaicin, clonazepam, alpha lipoid acid (ALA), vitamin supplement, zinc replacement and selective serotonin inhibitors; in addition, psychotherapy and behavioural therapy can also help to reduce or eliminate pain or burning (2,12,13).

In recent years, the use of biostimulating lasers has been proposed in several medical fields for treating chronic and acute pain conditions (14,15); promoting re-epithelialization, fibroblasts proliferation, collagen synthesis, increasing vascularity and decreasing the alterations in nerve impulse conduction. It is therefore clear to say that laser has demonstrated to have an anti-inflammatory and analgesic effect (16-22).

This paper aims to assess whether photobiomodulation therapy (PBMT) leads to an improvement in terms of both pain and quality of life in patients affected by Burning Mouth Syndrome.

Materials and Methods

Patients eligible for this study were recruited at the Department of Oral Medicine of Spedali Civili of Brescia (Italy) in the period from June 2015 to June 2018. Patients who had complained of oral pain or burning for more than 6 months were examined under standardized conditions, with artificial light, disposable retractors, and a mirror. Exclusion criteria were: age under 18 years, pregnancy, oral mucosal lesions, systemic diseases (hypertension, diabetes, anaemia, vitamin B12 or folic acid deficiency.), gastro-esophageal reflux, Sjogren’s syndrome, allergies, and hyposalivation. After the first visit, each patient underwent an oral swab. In case of positivity to Candidida or other microorganisms, the patient was excluded from the study.

Patients were then randomized by a computer code into two groups: group A, patients who underwent laser therapy, and group B, patients who received sham therapy (placebo) i.e. the device was turned on but the hand piece did not work. Laser/sham therapy was dispensed once a week for ten weeks. The researchers were blind to the randomised allocations.

The laser instrument used for this trial was K Laser Cube 3®; it was porTableand easy to handle. The laser was applied by a trained dentist and irradiated the most painful areas in the oral cavity, with discontinuous combined wavelengths between 660-970 nm, medium power 3.2 W (6.4 W pulsed at 50%), treatment time 3’51”, frequency 1-20000Hz, spot size 1cm2.

Dentists and patients wore appropriate protective eyewear and international safety procedures were followed.

- Pain Scoring

Burning mouth symptoms were evaluated through the Visual Analogue Scale (VAS). In this system, zero indicates no pain and ten indicates severe pain; patients were asked to select a number from 0 to 10 on a ruler with faces depicting the intensity of their pain. Pain assessment was performed at baseline, after each laser treatment, and at a month follow-up. Clinicians who performed the laser-treatments did not participate in the pain scoring.

- Quality of life related to Oral Health

The Italian version of the Oral Health Impact Profile questionnaire (OHIP-14) was also provided to evaluate the quality of life related to oral health (QLROH), during the first visit and once again after the last laser session. The short form of the OHIP-14 questionnaire, consisting of 14 questions related to oral health, was used to evaluate the quality of life (QoL) in relation to oral health. The Italian version of the OHIP-14 has already been validated (23) and summarises the following seven domains of impact on daily activities as a result of oral problems: functional limitation (domain 1), physical pain (domain 2), psychological discomfort (domain 3), physical activity (domain 4), psychological disability (domain 5), social disability (domain 6) and handicap or disability (domain 7).

The questionnaires were administered in interview form at baseline and at the end of each treatment session. Clinicians who performed the laser-treatments did not participate in the quality of life assessment.

- Statistical analysis

Data was recorded on spread-sheets and a statistical analysis was conducted using Stata® software for Mac; sex, age, localization were examined through descriptive analysis, including mean, standard deviation and percentiles.

Concordance or differences in the frequency distribution between the 2 groups were tested using Student t test; the variables were evaluated through split-plot analysis of variance (ANOVA) for repeated measurements as to identify possible associations between oral health and QoL. The results of VAS were compared using Wilcoxon test. A *p-value* <0.05 was considered statistically significant.

We hypothesised for there to be a 90% success rate at the end of the treatment for the group treated by laser and 60% for the placebo group. The minimum number of patients needed for the study, assuming alpha to be 0.05 and beta 0.10 (study power=90%), was calculated to be 78 (at least 39 participants per group).

- Ethical consideration

The study was approved by the local Ethical Committee (NP 1811-14) and all the patients were informed about the research and signed an informed consent before participating in the study.

## Results

**Figure 1:**
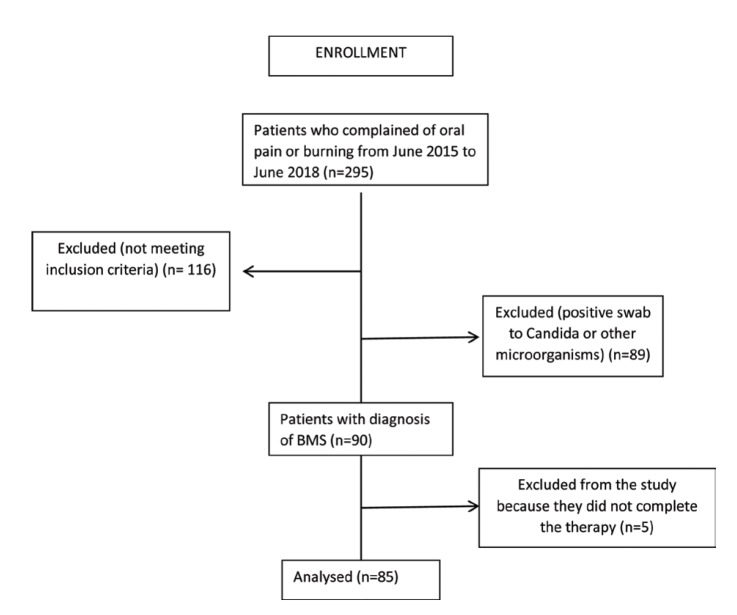
Enrollment.

A total of 90 patients were included in the study according to the enrolment criteria (45 patients per group); out of the sample that started the study, 85 completed it (Fig. 1).

Group A (laser treatment) was composed of 43 people, while group B (sham therapy) of 42 people. Demographic distribution and clinical data of the subjects are displayed in Table 1.

At baseline (T0), the VAS score was similar in the two groups (*p*=0.75). After the 5th therapy session (halfway through the full course of treatment), there was a reduction of the VAS mean; however, there still was not a statistically significant difference between group A and group B (*p*=0.6232). After the complete course of therapy, the patients treated with PBMT showed a significant decrease in symptoms (*p*=0.0008), which was maintained at the one-month follow-up (*p*=0.0005).

**Table 1:**
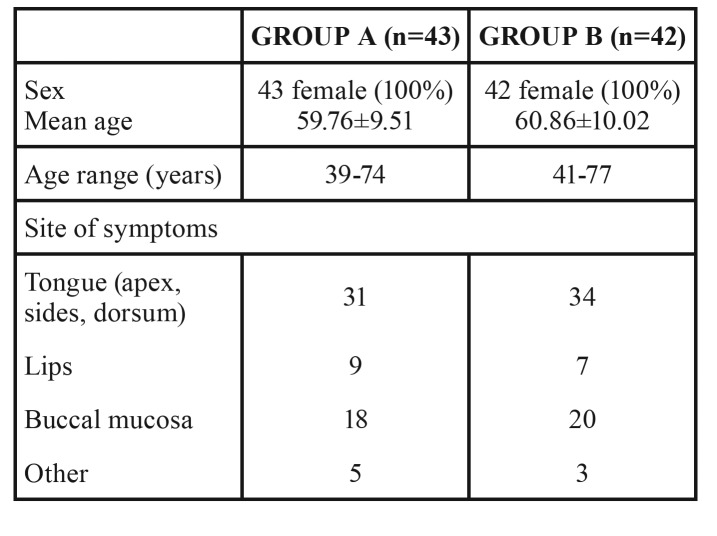
Demographic characteristics of the patients who completed the trial.

The scores for OHIP-14 index are displayed in Table 2. The bivariate analysis showed that the use of PBMT was statistically associated with an improvement in the quality of life related to oral health after the 7th week of treatment till the follow-up (Table 2).

**Table 2:**
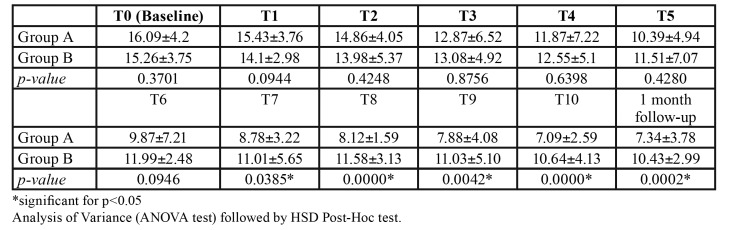
OHIP-14 scores (mean±SD) in group A and group B at baseline (T0), after every treatment and at one-month follow-up.

## Discussion

This randomized double-blinded prospective study demonstrates the efficacy of laser therapy in the treatment of Burning Mouth Syndrome.

In regards to the sample’s demographic characteristics, it is interesting to note that all the patients enrolled were female and middle-aged, upholding data reported in literature.

At the beginning of the trial, all patients complained of BMS symptoms, which reduced during the study period; both for VAS and for OHIP-14 index, cutback was significant only in the second half of the trial; this confirms that VAS improvement has a positive impact on quality of life.

Even if the exact mechanisms through which the laser works for pain relief are still not clear, it has been demonstrated that laser light has three main effects: analgesia, anti-inflammation and promoting wound healing (24,25). During its interactions with biological tissues, laser energy is converted into energy useful to cells (18); it induces an augmented production of mitochondrial ATP production, serotonin and endorphins. Moreover, local blood circulation, cellular proliferation and protein synthesis are increased. It is known that anti-inflammation and analgesia are connected to both an increase of peripheral endogenous opioids and a decrease of pro-inflammatory cytokines and free oxygen radicals (16). A recent trial of Pezelj-Ribaric, *et al*. (20) demonstrated that salivary levels of TNF-alpha and IL-6 in patients affected by BMS significantly reduced after 4 weeks of treatment with low level laser therapy (LLLT), with clinical improvement of BMS symptoms. Since the salivary levels of pro-inflammatory cytokines can be used as biological indicators of BMS (26), we can speculate that laser therapy with its mechanism of action can be useful in disease management, as suggested in the present trial.

This double-blind study was conducted with a treatment and a placebo arm; a recent review of Kuten-Shorrer, *et al*. (27) found that, even if a lot of randomized controlled studies were conducted about BMS treatment, the selection of a placebo was inconsistent. It is known that BMS is not always only a physical disease but also a psychological problem; this leads to evaluate that the placebo effect must be strongly considered when investigating the improvement of symptoms. The cited review reported a mean placebo response as a fraction of treatment response of about 72%, which can be considered a very strong placebo response. In literature, about 60% of studies about BMS therapy showed improvement in the treated sample but a positive response to the placebo ranging from 15% to 74% (28,29) was also exhibited. Given the subjective nature of BMS symptoms and the strong placebo response, Kuten-Shorren, *et al*. (27) suggested to include a third arm in the BMS treatment randomized control trials (RCTs) made up of a no-treatment waitlist control group; the absence of this type of arm could be considered a weak point of the present survey.

The positive results of our investigation support the choice of PBMT for treating BMS symptoms. Previous studies have been conducted, studying the influence of varying laser device parameters such as power, wavelengths and number of sessions, but in general PBMT resulted in a significant improvement of the disease (14,21,30,31). More RCTs studies are needed to define specific device parameters and protocols to be applied in the everyday clinical management of BMS.
